# What’s hot in neonatal respiratory monitoring?

**DOI:** 10.1038/s41390-024-03746-8

**Published:** 2024-11-17

**Authors:** Emma Williams, Theodore Dassios

**Affiliations:** 1 Department of Women and Children’s Health, School of Life Course Sciences, Faculty of Life Sciences and Medicine, King’s College London, London, UK; 2 Neonatal Intensive Care Unit, University of Patras, Patras, Greece

## Abstract

- Respiratory monitoring is an essential part of routine clinical care of the newborn.
- Recent technological developments have improved respiratory monitoring and allowed for a two-way interaction between the monitored parameter and the level of the provided respiratory support.
- We herein discuss applications of monitoring by neurally adjusted ventilatory assist, closed-loop oxygen control, tidal capnography, lung ultrasound, diaphragmatic electromyography and lung magnetic resonance imaging.

Advances in the respiratory management of ventilated newborns, including volume-targeted and high-frequency ventilation, increased use of non-invasive ventilation and the application of less invasive methods to administer surfactant have contributed to the increased survival of even the tiniest and most immature neonates. This comes with the cost, however, of higher rates of chronic respiratory morbidity. Individualised, responsive and high-precision respiratory monitoring becomes thus a pressing priority in the care of these infants, aiming to minimise lung injury and promote growth and lung recovery. A number of novel methods and technological advancements have emerged over the past years, which allow for an interaction between a chosen monitored parameter with the level of the provided respiratory support, enabling a personalised and dynamic approach to neonatal respiratory management. The aim of this commentary is to highlight recent advancements in areas of neonatal respiratory monitoring which have significant potential to improve clinical outcomes and minimise ongoing lung injury during the initial phase of ventilation and stabilisation and during the later stage of evolving or established chronic respiratory disease.

Neurally Adjusted Ventilatory Assist (NAVA) is a mode of invasive or non-invasive ventilation with a sophisticated triggering mechanism, which operates based on continuous readings of the electromyographic signal of the diaphragm (Edi) and subsequently delivers proportionate inflation pressures in response to the Edi signal at a breath-by-breath frequency. NAVA has been proven to be particularly useful in the successful extubation of infants with severe bronchopulmonary dysplasia (BPD): in a retrospective multi-centre study of infants with severe BPD, NAVA was successful in 67% in achieving respiratory stability at a lower level of ventilatory support, including extubation to non-invasive positive pressure ventilation or support with a home ventilator.^1^ Although NAVA is primarily a mode of ventilation, the provided personalised and proportionate support is an example of integrating advanced monitoring (Edi is the monitored parameter) and individualised support (the delivered inflation pressure is a product of the Edi).

Another such technological advancement is closed-loop automated oxygen control (CLAC), where the provision of the fraction of inspired oxygen is individually tailored to the continuous synchronous reading of transcutaneous oxygen saturation (SpO_2_) with a view to maintain the SpO_2_ within a specific predefined range. This technology has been reported to decrease the amount of time spent below and above the target SpO_2_ ranges and to decrease the number of manual adjustments made over a specified period of care.^2^ While CLAC could plausibly be associated with decreased complications with an oxidative pathophysiological background, the measured impact of CLAC on clinical outcomes such as retinopathy of prematurity (ROP), BPD and mortality has not yet been reported, possibly due to the complicated multivariable aetiology of respiratory disease and interrelated co-morbidities in extreme prematurity.

Similarly, and given the important impact of the fluctuations of carbon dioxide (CO_2_) on the development of intracerebral haemorrhage and ischaemia in newborns, it is possible that closed-loop ventilation systems could also be developed in the future. These systems could adjust the delivered pressure or tidal volume in response to continuous readings of tidal CO_2_, aiming towards better CO_2_ control and less fluctuation to extreme high and low CO_2_ values. The current technology to monitor tidal CO_2_ has improved over the past years, and the incorporation of tidal capnography in clinical neonatology has the potential to improve clinical outcomes and reduce the frequency of blood gas sampling. Furthermore, tidal capnography could provide insights into the pathophysiology of acute and chronic neonatal respiratory disease, as the evolving difference between the arterial and end-tidal CO_2_ can provide real-time information on the anatomical and alveolar dead space which could in turn guide respiratory management by delivery of alveolar volumes above the dead space ventilation, thus providing sufficient tidal volumes for gas exchange.^3^

Surface diaphragmatic electromyography (sEMG_Di_) has also seen a renewed interest over the past years with the emergence of new devices with adhesive pads which are easier to apply on the chest and are less complicated to interpret, compared to the previously used trans-diaphragmatic catheters and amplifiers. sEMG_Di_ has been used to describe that the effect of intravenous caffeine on diaphragmatic contractility in ventilated preterm infants reaches a peak thirty minutes after intravenous loading.^4^ This information can suggest an optimal time window for extubation.

Significant clinical interest and focused training on lung ultrasonography have also enhanced the uptake of this method as a sequential point-of-care monitoring tool in neonatal intensive care. Lung ultrasound can assist in the diagnosis of disease states such as neonatal respiratory distress syndrome (RDS), transient tachypnoea of the newborn, pneumothorax and for the differential diagnosis of atelectasis or fluid collection which are not always distinguishable by chest radiography. Recent scoring methods have been developed which allow for the quantification of the severity of neonatal RDS and can assist the physician during acute clinical management and in follow-up.^5^

Improved care and increased survival of extremely preterm infants highlights the need for the optimisation of ventilation monitoring in these infants, with a view to maximise alveolar recruitment while avoiding excessive ventilation and overdistention, which could cause devastating lung injury and acute perfusion impairment. Electrical impedance tomography (EIT) is such a monitoring technology which could assist in fine-tuning alveolar ventilation by real-time monitoring of optimal recruitment and ventilation/perfusion (V_A_/Q) matching. EIT operates by detecting differences in tissue conductance in response to an electrical current to visualize changes in lung aeration. This modality can be used as a cot-side tool to monitor regional ventilation distribution and end-expiratory lung volume. EIT is a non-invasive, radiation-free method which requires the placement of a belt containing non-sticky electrodes placed around the chest and can detect and monitor regional changes in ventilation attributed to pneumothorax, atelectasis or different infant positions. EIT could also be used to individualise respiratory care and guide optimal lung recruitment at varying pressure levels while on nasal continuous positive airway pressure, conventional and high-frequency ventilation.^6^

Longer-term monitoring of respiratory function in the newborn could also be achieved by non-volitional methods such as the forced oscillation technique (FOT). FOT is a non-invasive method to assess lung function and respiratory mechanics at the cot side and can be applied during regular spontaneous breathing. FOT works by applying small amplitude pressure stimuli at the airway opening and measuring the corresponding mechanical response of the lungs measured mainly as reactance and resistance. A higher reactance of the respiratory system measured on the seventh day of life in preterm infants has been associated with a longer duration of respiratory support and was higher in infants who developed BPD – suggesting FOT could improve the prediction of BPD when added to standard demographic parameters such as the gestational age and birth weight.^7^

Given the concerns regarding radiation exposure with serial computerised tomography scans, recent advances in lung Magnetic Resonance Imaging (MRI) have highlighted this non-ionizing modality as one with significant potential in advanced sequential respiratory monitoring in the newborn. Quiet-breathing ultrashort lung MRI has been applied in the neonatal unit and MRI-derived BPD severity scores predicted the need for respiratory support at discharge from neonatal care. The MRI scores were stronger predictors of the duration of respiratory support compared to clinical data such as gestational age or birth weight. Additionally, neonatal lung MRI could serially assess structural abnormalities in BPD, characterise disease trajectories and potentially individualise care and classify BPD infants to separate disease phenotypes.^8^ Future applications of neonatal lung MRI include functional mapping of V_A_/Q matching by simultaneous acquisition of ventilation and perfusion images in a single scan with potential applicability in individualising respiratory care and in tailoring care aimed at targeting different lung regions in the same infant.^9^

Aside from imaging, functional methods have also been developed to monitor V_A_/Q matching and ongoing respiratory disease in newborns. We have recently described a non-invasive method to estimate the alveolar surface area (S_A_) in extremely preterm-born infants and reported that the S_A_ was lower in infants who required supplemental oxygen at home compared to those who did not and was significantly inversely correlated with the duration of inpatient oxygen therapy.^10^ We further reported that the S_A_ was also significantly associated with the total duration of home oxygen therapy and was significantly lower in infants with abnormal neurodevelopment at two years of age compared to infants without or with mild impairment at follow-up.^10^ The S_A_ could thus be used as a possible non-invasive monitoring biomarker for neonatal respiratory disease during inpatient neonatal care and could be useful in longer-term follow-up and in planning for home oxygen.

In conclusion, recent advances in neonatal respiratory monitoring offer the potential of enhanced, individualised respiratory care and allow for interaction between the monitored parameter and the level of delivered respiratory support.
